# Apneic Tracheostomy in COVID-19 Patients on Veno-Venous Extracorporeal Membrane Oxygenation

**DOI:** 10.3390/membranes11070502

**Published:** 2021-06-30

**Authors:** Matteo Rossetti, Chiara Vitiello, Valeria Campitelli, Raffaele Cuffaro, Claudia Bianco, Gennaro Martucci, Giovanna Panarello, Federico Pappalardo, Antonio Arcadipane

**Affiliations:** Department of Anesthesia and Intensive Care, IRCCS-ISMETT, UPMC Italy, 90133 Palermo, Italy; mrossetti@ismett.edu (M.R.); cvitiello@ismett.edu (C.V.); vcampitelli@ismett.edu (V.C.); rcuffaro@ismett.edu (R.C.); cbianco@ismett.edu (C.B.); gmartucci@ismett.edu (G.M.); gpanarello@ismett.edu (G.P.); fpappalardo@ismett.edu (F.P.)

**Keywords:** COVID-19, SARS-CoV-2, tracheostomy, veno-venous ECMO, ARDS, respiratory faiulure, safety, infection control

## Abstract

COVID-19 creates an impressive burden for intensive care units in terms of need for advanced respiratory care, with a huge number of acute respiratory distress syndromes (ARDS) requiring prolonged mechanical ventilation. In some cases, this proves to be insufficient, with a refractory respiratory failure calling for an extracorporeal approach (veno-venous ECMO). In this scenario, most of these patients need an early tracheostomy procedure to be carried out, which creates the risk of distribution of aerosol particles, possibly leading to personnel infection. The use of apneic tracheostomy has been proposed for COVID-19 patients, but in case of ECMO it may produce lung derecruitment, severe hypoxemia, and sudden worsening of respiratory mechanics. We developed an apneic tracheostomy technique and applied it in over 32 patients supported by veno-venous ECMO. We present data showing the safety and feasibility of this technique in terms of patient care and personnel protection. Gas exchange and pH did not show statistically significant changes after the tracheostomy, nor did respiratory mechanics data or the need for inspiratory pressure and FiO_2_. The use of apneic tracheostomy was a safe option for patient care during ECMO and reduced the possibility of virus spreading.

## 1. Introduction

The morbidity, mortality, and high rate of transmission of SARS-CoV-2 have caused an unprecedented worldwide health crisis. Although the majority of patients show mild symptoms, approximatively 10–17% of those hospitalized eventually develop severe acute respiratory failure requiring admission to an intensive care unit for mechanical ventilation and eventually veno-venous extracorporeal membrane oxygenation (V-V ECMO) [1,2]. The mechanical ventilation during ECMO is usually prolonged, and tracheostomy may facilitate patient management [3]. In patients affected with COVID-19, mechanical ventilation has proven to be particularly prolonged; consequently, tracheostomy is considered quite frequently. Despite several positive general considerations (like the potential reduction of sedatives and reduced incidence of ventilator-associated pneumonia or laryngeal stenosis), percutaneous tracheostomy is also considered an aerosol-generating procedure which breaks the continuity of the breathing circuit, with derecruitment, loss of positive end-expiratory pressure (PEEP), and desaturation. Minimizing the exposure of health care personnel to aerosol-generating procedures is critical in the COVID-19 era [4,5,6]. In fact, front-line healthcare workers exposed to the aerosolized virus may succumb to the disease, which has consequences for the individual and also at the healthcare system level, since affected workers may result in further personnel shortages.

In this context, the use of tracheostomy has been reconsidered in COVID-19 patients, and the concepts of patient selection and careful evaluation of the correct timing for the procedure have been highlighted.

Recently, a case series of mechanically ventilated COVID-19 patients showed the feasibility and safety of a modified technique for tracheostomy based on apnea, in order to minimize the risk of viral infection and ensure the safest pathway for the patients [7].

Patients who require V-V ECMO routinely need tracheostomy; these are the sickest patients and require tracheostomy early and systematically [8,9,10,11]. Turning the ventilator to ‘standby’ mode and allowing decompression of the breathing circuit might lead to de-recruitment and desaturation, but aerosolization is minimized. Since patients on ECMO are in an unstable balance between the partial support and native lung gas exchanges, the apnea time for tracheostomy might theoretically cause derecruitment and severe desaturation. However, the extracorporeal support can be exploited by the operators to approach the procedure more safely, as apnea may be maintained via extracorporeal gas exchange [12,13,14].

We report an experience of apneic tracheostomy in a consecutive series of COVID-19 patients on V-V ECMO, showing that clinically relevant ECMO-related measures remained unchanged before and after the procedure, indicating the safety of the apneic approach alongside the advantage of reducing virus transmission to healthcare professionals.

## 2. Materials and Methods

We performed a single-center observational cohort study of patients with COVID-19 admitted between September 2020 and April 2021 to the Mediterranean Institute for Transplantation and Highly Advanced Therapies (ISMETT) COVID-19 Intensive Care Unit (ICU) for respiratory failure requiring V-V ECMO support. All data were routinely recorded on the hospital electronic chart system and then retrospectively analyzed. Institutional review board approval was obtained. Demographic and clinical data were collected before and 24 h after the tracheostomy, including duration and type of ventilation, ECMO parameters, blood gases, and complications.

### 2.1. Tracheostomy Procedure, Anticoagulation Management, and ECMO Management

All the procedures were performed at the patient’s bedside in the ICU via the percutaneous Ciaglia Blue Rhino technique (Ciaglia Blue Rhino, Cook Critical Care, Bloomington, Illinois) applied by four attending intensivists wearing personal protective equipment (PPE) as per protocol; at least one nurse was at the bedside for support during the procedure. All patients were paralyzed to minimize coughing and aerosolization during airway manipulation.

Patients were positioned supine with a transverse rolled towel beneath the scapulae to augment neck extension. An ultrasound of the neck was performed before the procedure to identify possible vascular alterations.

Before the procedure, personnel involved each donned an N95 respirator mask, a disposable scrub cap or bouffant, an isolation gown, a surgical gown, a face shield, inner and outer gloves, and knee-high shoe covers. 

Unfractionated heparin was stopped at least 2 h before and was usually restarted approximately 4 h after the procedure in the absence of concerning local bleeding. Platelet count was mandated at >50,000 per µL, and the procedure was postponed if the value was below the desired value.

Before starting the procedure, the intravascular volume was optimized by packed red blood cell transfusion in the case of an hemoglobin value <8 g/dL or by the administration of albumin 5% (usually 250 to 500 mL) to achieve the constant maximum expected blood flow for the patient’s body surface. After the maximum blood flow test, the sweep gas flow was retested. The ECMO flows (pump and sweep) remained unchanged throughout the whole procedure, and blood flow was maintained to maintain SpO_2_ > 85%. The first apneic pause was performed to introduce the fiber optic bronchoscope through the port of the airway circuit connector; the trachea was visualized and the bronchi were inspected for obstruction by mucus or blood, which were then cleared before further maneuvers. The whole procedure was performed using single-use bronchoscopes (Bronchoflex^®^ Vortex, AccessVision, Tours, France).

The procedure then began and the ventilator was set to standby until the tracheostomy cannula position was checked. A blood gas and chest X-ray was routinely done afterwards. 

Bleeding was defined as the need for further bronchoscopy or the need for blood transfusion in the following 12 h.

### 2.2. Surveillance of Healthcare Workers 

All staff members of the COVID-19 ICU at ISMETT were routinely monitored with nasopharyngeal swab and serology test every 21 days and in case of any ‘COVID-like’ symptoms. Vaccination was started at the end of December 2020 and completed by the 24th of January 2021.

### 2.3. Statistical Analysis

Categorical variables are reported as number and percentage, while continuous variables are reported as mean and standard deviation. Continuous variables between periods before tracheostomy and after tracheostomy were assessed using *t*-tests.

Data managing and descriptive analyses were done with SAS 9.4 statistical software, and a *p* value of <0.05 was considered the cut-off for statistical significance.

## 3. Results

Thirty-two COVID-19 patients with acute respiratory failure treated with V-V ECMO were enrolled in the study. Patients were predominantly male (28 patients, 87.5%); the average age was 51 ± 11 years old (range 22–67), confirming the wide spectrum of patients affected by the most severe forms of COVID-19. The average duration of mechanical ventilation before tracheostomy, defined as the time from first intubation to tracheostomy, was 11 ± 6 days (range of 5–25); the average duration of the V-V ECMO run before tracheostomy was 8 ± 3 days. The demographic characteristics of the population are shown in Table 1.

Blood gas analysis, mechanical ventilation, and ECMO settings before and after the apneic procedure are reported in Table 2. Notably, the patients were on ‘full support’ at the time of the procedure, as shown by average ECMO blood flow of 4 L/min, sweep gas flow of 6 L/min, and PEEP 12 cm H_2_O with FiO_2_ 0.5; mean PaO_2_ was 68.6 ± 23.2 mmHg. No complications were recorded during the procedures, which were accomplished without the need for any intervention to restore oxygen tension or hemodynamics; clinical values after the procedures were unchanged. In particular, the value of PaO_2_ after the procedure was unchanged (*p* value of 0.21). Increase of blood flow support to compensate for serious desaturation was not required (BF pre 4 ± 1.4 lpm, BF post 4 ± 1.2 lpm, *p* = 0.48) (Table 2). No pneumothorax was observed. Six cases of airway bleeding were observed late in the course of care.

We did not observe any SARS-CoV-2 infection among the operators and healthcare professionals of the COVID-19 ICU.

## 4. Discussion

The role of tracheostomy in COVID-19 patients is still under debate, as the procedure is potentially linked to an augmented risk of viral spreading through generation of aerosol particles [15]. There are different strategies to reduce the abovementioned risk, for example the use of neuromuscular blocking agents to minimize the cough reflex and the execution of the procedure by highly specialized teams in order to reduce the duration time and the risk of loss of the airway. All personnel in the room must wear personal protective equipment (PPE) and observe the institutional policies regarding the use of PPE during aerosol-generating procedures. The number of people in the room should be limited to those absolutely necessary, but a minimum of three medical personnel must be present [16,17]. As most patients no longer exhibit viral shedding by 21 days from symptom onset, an alternative approach might be the avoidance of tracheostomy before 21 days of endotracheal intubation [18,19]. However, the contemporary approach to airway management in V-V ECMO suggests an earlier timing for the procedure. Shield strategies for the operators and personnel are common practice in this scenario; however, multiple issues have been raised with this approach over the last year of experience with COVID-19. First, the manual and technical skills of personnel are decreased by their gowns, multiple gloves, and face masks; second, the high workload requires multiple operators to perform procedures which cannot be assigned only to experienced colleagues; lastly, safety has to be strictly guaranteed both for patients and healthcare professionals [20].

The main finding of the present investigation was that tracheostomy can be safely and successfully performed in patients with COVID-19 and V-V ECMO during apnea. To the best of our knowledge, our study describes for the first time the feasibility and safety of this strategy focusing just on V-V ECMO, and showed neither respiratory deterioration nor airway emergency, with low risk of complications in the following 24 h [21,22]. Recently, Weiss et al. elegantly described the efficacy of an apneic phase during the same procedure of percutaneous tracheostomy performed by a skilled and trained tracheostomy team [7]. In fact, this initiative was safe from both the patient and the personnel perspectives. However, looking at the data, just 25% of patients were severely sick and supported by ECMO. Consequently, the data cannot be directly generalized to the ECMO population, giving the potential for extreme patient selection.

In our experience, V-V ECMO allowed safe apnea and did not affect pulmonary mechanics; indeed, no significant clinical signs of derecruitment of the lung were observed, confirming the peculiar features of COVID-19 ARDS in terms of lung elastance and compliance. A system for assessing readiness for tolerating apnea is an apnea trial: it was recently described and recommended by an international, multidisciplinary panel of tracheostomy experts [9,10]. The apnea trial consists of pre-oxygenation followed by a trial of apnea, simulating the physiological conditions to which the patient would be exposed during the percutaneous procedure. This expedient has great value in patients for whom their functional reserves must be established [10]; on the other hand, a failed trial could worsen an already critical situation: hypercapnia and hypoxia could deteriorate and further hypercapnia could generate acidosis, which may lead to poor cardiac contractility or arrhythmias. In addition, permissive apnea leads to worsening hypoxia, and these patients frequently need a long time to recover, given their poor pulmonary function [11]. Our approach presents a crucial difference, namely the presence of extracorporeal support for gas exchange. This means, in short, that patients are able to tolerate the apneic phase very well. 

The operators were not infected over the study period. No infective events among the operators were recorded, thus leading to the conclusion that the discontinuation of mechanical ventilation throughout the whole procedure leads to the protection of the whole team without bearing the risk of desaturation of the patient.

Although this study illustrates a large series of cases focused on V-V ECMO patients infected with COVID-19, it has several limitations. First, this series is based on a single center’s experience. Second, and as a consequence of the first potential bias source, there was no control group for either the timing of tracheostomy or the technique. Finally, we present only the early feasibility of this approach during ECMO support; the long-term outcomes should still be evaluated, with the consideration that the tracheostomy procedure during ECMO is still debated for it concrete usefulness.

In conclusion, apneic tracheostomy is feasible during V-V ECMO for COVID-19 and allows for a timely tracheostomy. ECMO support, instead of being a contraindication to tracheostomy, might facilitate the performance of this procedure in terms of safety for operators. During the short apneic time, the clinically relevant ECMO-related measures remained unchanged before and after the procedure, indicating the safety of the apneic approach with the advantage of reducing virus transmission to healthcare professionals.

## Figures and Tables

**Table 1:** Baseline characteristics and demographic features. Dichotomous variables are reported as count and percentage; continuous variables are expressed as mean ± standard deviation. (COPD: chronic obstructive pulmonary disease; RPM: round per minute; FiO_2_: inspiratory oxygen fraction; MV: mechanical ventilation; SD: standard deviation).

Demographic Data	Mean ± SD or N (%)
Age, years	51 ± 11.0
Male gender, *n* (%)	28 (87.5)
Height, cm	173 ± 5.0
Weight, kg	95 ± 13.3
Obesity, *n* (%)	21 (65.6)
Diabetes, *n* (%)	5 (15.6)
Hypertension, *n* (%)	12 (37.5)
Smoker, *n* (%)	4 (37.5)
COPD, *n* (%)	1 (3.0)
Asthma, *n* (%)	1 (3.0)
Chronic renal failure, *n* (%)	1 (3.0)
Chronic liver disease, *n* (%)	1 (3.0)
Chronic heart failure, *n* (%)	1 (3.0)
Immunosuppression, *n* (%)	1 (3.0)
** ECMO parameters **	
Blood flow, L/min	4.0 ± 1.4
Sweep gas flow L/min	6.0 ± 2.0
Revolution per minute, rpm	2743 ± 623
ECMO days before procedure	8 ± 3.0
** Ventilator settings **	
Inspiratory pressure, cmH2O	24 ± 5.0
Respiratory rate, breaths/min	10.0 ± 2.6
FiO_2_	0.5 ± 0.16
MV days before procedure	11 ± 6.0

**Table 2:** Arterial blood gas analyses, mechanical ventilation, and ECMO data before and after apneic tracheostomy (where ABG: arterial blood gas analyses; PaCO_2_: arterial partial pressure of carbon dioxide; PaO_2_: arterial partial pressure of oxygen; MV: mechanical ventilation; Pinsp: inspiratory pressure; RR: respiratory rate; PEEP: positive end-expiratory pressure; FiO_2_: inspiratory oxygen fraction; RPM: round per minute; BF: ECMO blood flow; SGF: ECMO sweep gas flow). *p* Values refer to paired *t*-tests.

	Before Tracheostomy	After Tracheostomy	*p* Value
Mean Values			
**ABG**			
pH	7.43 ± 2.9	7.43 ± 2.5	0.34
PaCO_2_, mmHg	44.5 ± 11.0	44 ± 7.0	0.42
PaO_2_, mmHg	68.6 ± 23.2	66.75 ± 23.6	0.21
**MV**			
Insp. Pressure, cm H_2_O	24 ± 5	24 ± 2.8	0.47
RR, breaths/min	10 ± 2.6	10 ± 1.8	0.45
PEEP, cm H_2_O	12 ± 3.5	12 ± 2.8	0.5
FiO2	0.5 ± 0.16	0.5 ± 0.1	0.2
**ECMO**			
RPM, rpm	2742 ± 622	2645 ± 640	0.32
BF, lpm	4 ± 1.4	4 ± 1.2	0.48
SGF, lpm	6 ± 2.0	6 ± 1.9	0.38

## Data Availability

The dataset used and analyzed are available from the corresponding author on reasonable request.
